# The Impact of Family Functioning on College Students’ Loneliness: Chain-Mediating Effects of Core Self-Evaluation and Problematic Mobile Phone Use

**DOI:** 10.3389/fpsyg.2022.915697

**Published:** 2022-07-06

**Authors:** Ling Qian, Die Wang, Min Jiang, Wei Wu, Congying Ni

**Affiliations:** ^1^School of Business, Jinhua Polytechnic, Jinhua, China; ^2^College of Teacher Education, Zhejiang Normal University, Jinhua, China

**Keywords:** family functioning, loneliness, core self-evaluation, problematic mobile phone use, college students

## Abstract

**Purpose:**

To examine the influence of family functioning on college students’ loneliness and the mediating effects of core self-evaluation and problematic mobile phone use.

**Methods:**

Family Function Scale, Core Self-evaluation Scale, Problem Mobile Phone Use Scale, and Loneliness Scale were used to investigate 8,524 college students.

**Results:**

(1) Family functioning positively predicted core self-evaluation (β = 0.43, *p* < 0.001) and negatively predicted loneliness (β = −0.21, *p* < 0.001); (2) Core self-evaluation negatively predicted problematic mobile phone use and loneliness (β = −0.34, *p* < 0.001; β = −0.50, *p* < 0.001); (3) Problematic mobile phone use significantly positively predicted loneliness (β = 0.05, *p* < 0.001); (4) Core self-evaluation and problematic mobile phone use showed a significant chain-mediation effect between family functioning and loneliness (β = −0.01, *p* < 0.001).

**Conclusion:**

The results are helpful to comprehend the producing mechanism of loneliness and provide a theoretical basis for the intervention of loneliness.

## Introduction

Loneliness refers to an unpleasant psychological experience when there are differences between the desired social relationship and the actual social relationship network in quality (such as the loss of intimate relationship) or quantity (such as too few friends) (Smoyak, 1984). Long-term loneliness cannot only lead to painful emotions and depression (Zhang et al., 2019), but also lead to suicidal tendencies (Li et al., 2018), anti-social behavior, hostility, and sleep disorders (Li et al., 2016). At the annual meeting of the American Academy of Child and Adolescent Psychiatry in 2020, it was reported that lonely young people were three times more likely to suffer from depression in the future, and the impact of loneliness on mental health might last at least 9 years. Domestic research showed that more than 50% of college students experienced loneliness (Cheng and Zhao, 2017), and loneliness became a common problem among college students (Tang et al., 2014).

Psychological research on loneliness has a history of several decades. From the positive-negative dimension, Moustakas (1961) divided loneliness into existential loneliness and loneliness anxiety. Existential loneliness is a positive growth experience of self-confrontation, which is less common in lonely people; loneliness anxiety is a negative experience caused by “basic alienation between people” and dominates the lives of lonely people (Russell et al., 1980). From the perspective of social deprivation, Weiss (1987) divided loneliness into emotional loneliness and social loneliness. Emotional loneliness stems from the absence of a private, intimate relationship or attachment, which is a more painful form of isolation; social loneliness stems from the absence of a social connection or sense of belonging to a group, which is a mixture of feelings of rejection or non-acceptance and boredom. This theory has been validated by several researchers in the field of measurement (Russell et al., 1984; Vincenzi and Grabosky, 1987). Ditommaso and Spinner (1993) expanded emotional loneliness to two subtypes: intimate loneliness and familial loneliness. Intimate loneliness refers to the lack of intimate romantic partners, while family loneliness refers to the lack of family members. Starting from the dimension of duration, Young (1982) divides loneliness into three types: transient loneliness, situational loneliness, and chronic loneliness. Transient loneliness refers to some transient, an occasional feeling of loneliness. Situational loneliness is when some specific change occurs that disrupts the original satisfying relationship, such as moving to new a place. Situational loneliness can trigger extremely painful experiences. Chronic loneliness is a condition in which a person has not had a satisfactory social relationship for more than 2 years. When situational loneliness lasts for a long time, it will turn into chronic loneliness. From the perspective of intervention, the greatest attention should be paid to how to prevent situational loneliness from becoming a serious and long-term experience.

In recent years, previous studies have examined the impacts of family cohesion (Yang et al., 2016) family atmosphere (Yang et al., 2013), parent-child relationship (Fan and Wu, 2020) and other family factors on loneliness, but less attention has been paid to the role of family function on loneliness. In addition, most of the existing studies focused on the impact of family on the loneliness of primary and secondary school students (Xing and Chi, 2003; Liu et al., 2018), but few studies focused on the role of family functioning on college students. According to the ecosystem theory, the family is the most direct and lasting micro-system in the source of individual development influence (Pomerantz et al., 2008). Studies have shown that family functioning still has a profound impact on college students (Fang et al., 2006). Family functioning refers to the effectiveness of family rules, family communication, emotional connections, and coping with external events among family members in the family system (Olson, 2000). According to the theory of family function, the normal operation of basic family functions can promote the development of individual mental health and enhance the internal emotional connection of family members (Olson, 2000). Previous studies have found that family functioning is a direct predictor of loneliness (Deng and Zheng, 2013; Wang et al., 2019). Although many studies have shown that family functioning is related to individual loneliness, it is not clear through which mechanism family functioning affects college students’ loneliness. Therefore, this study will further explore the internal mechanism of family function affecting loneliness, and provide reference methods and paths for the intervention of loneliness.

Family functioning not only directly affects loneliness, but also indirectly affects college students’ loneliness through core self-evaluation. Core self-evaluation refers to the most basic evaluation of an individual’s ability and value, including general self-efficacy, self-esteem, neuroticism, and locus of control (Judge et al., 1997). Some studies suggested that the attitude toward self-internalized by individuals in childhood would continue into adulthood (He, 2015), and individuals with better family function had positive self-concept (Tian, 2012). Evaluation theory holds that emotion is a form of subconscious evaluation of objects, individuals or events by their perceived values, needs or commitments (Lazarus and Folkman, 1984). Previous studies have shown that core self-evaluation had a significant impact and predictive effect on mental health (Liu and Jiang, 2019). Core self-evaluation positively predicted positive emotions and negatively predicted negative emotions (Li and Sun, 2007). College students with high core self-evaluation were emotionally stable and optimistic (Da, 2002). Negative self-evaluation would lead to more flinch, avoidance and other behaviors (Yang, 2019). It is concluded that individual core self-evaluation is influenced by family functioning and is related to individual loneliness. Based on this, this study proposed that core self-evaluation played a mediating role between family functioning and loneliness (Hypothesis 1).

Family functioning not only indirectly affects loneliness through core self-evaluation, but also indirectly affects loneliness through problematic mobile phone use. Problematic mobile phone use refers to uncontrolled and excessive mobile phone use that adversely affects an individual’s daily life (Billieux, 2012). The theory of family function points out that a well-functioning family can effectively promote the mental health of individuals (Olson, 2000). Studies have shown that parenting style and social support had significant impacts on mobile phone dependence behavior, and individuals with insufficient social support in real life were more likely to form mobile phone dependence behavior (Zhao, 2020). Mobile phone addiction would have a negative impact on the individual’s physiology, psychology, and behavior (He and Xia, 2021). A meta-analysis conducted by Zhang and Li (2020) on the relationship between loneliness and mobile phone addiction showed that there was a moderate positive correlation between mobile phone addiction and loneliness. Based on the above analysis, this study proposed that problematic mobile phone use played a mediating role between family functioning and loneliness (Hypothesis 2).

In the past four decades, researchers mainly explored the influencing factors of loneliness from the aspects of environment and individual (Wang et al., 2015; Pu, 2020). In addition to the direct effect of family functioning on loneliness, family functioning, as an external factor, may also affect loneliness through individual internal factors and individual behavior. Core self-evaluation (intra-individual characteristic) and problematic mobile phone use (individual behavior) are important factors. According to the cognitive behavioral model of pathological Internet use (Chen et al., 2020), cognitive symptoms of pathological Internet use can lead to emotional or behavioral symptoms, and individual cognition or thought is the main source of abnormal behavior. Therefore, the problem behavior of problematic mobile phone use mainly resulted from the individual’s internal cognition. Studies have shown that adolescents with low core self-evaluation were more likely to become addicted to smartphones (Chen et al., 2020). Core self-evaluation was a negative predictor of Internet addiction (Lu and Zheng, 2011). Family, as the most persistent micro-system in the source of individual development influence, not only affected individual core self-evaluation (Tian, 2012), but also had a negative correlation between family function and mobile phone dependence (Shi et al., 2020). To sum up, family function may affect core self-evaluation, and then affect the use of problematic mobile phones, which eventually has an impact on individual loneliness. Accordingly, we assumed that core self-evaluation and problematic mobile phone use played a chain mediating role between family functioning and loneliness (Hypothesis 3).

**FIGURE 1:**
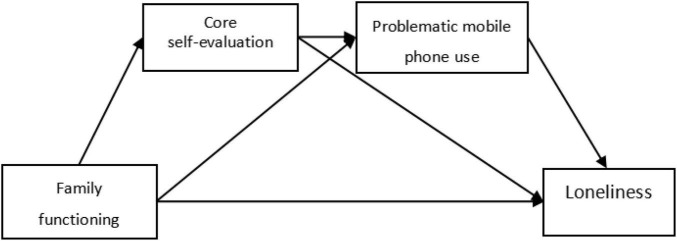
Hypothetical model paths of family functioning, core self-evaluation, problematic mobile phone use, and loneliness.

Based on the above discussion, this study aimed to examine the impact of family functioning on college students’ loneliness, and the sole/chain mediating role of core self-evaluation and problematic mobile phone use. Considering that gender may affect loneliness (Pu, 2020), gender is included in the model as a control variable. Based on the existing theory and empirical evidence, this study proposed the following hypothetical model (see Figure 1).

## Materials and Methods

### Participants

Using cluster sampling method, 8,524 students in Zhejiang and Anhui provinces were selected as the subjects, with an average age of 19.06 ± 1.03 years, including 3,577 boys (41.96%), and 4,947 girls (58.04%).

### Procedures

The data collection was led by well-trained psychology graduate students and supervised online by the head teacher of each class. Before starting the formal investigation, written informed consent from the school, the teacher, and the participants was obtained. Participants were informed that they could discontinue or drop out freely. The researchers clearly emphasized the confidentiality of the study results that any information provided by participants would not be disclosed to anyone.

### Measures

The family function scale revised by Zou was used to measure student’s family functioning (Cheng and Zou, 2011). The scale is of a single dimension, with a total of 6 items. A 5-point scoring system was used, from “completely inconsistent” to “completely consistent,” such as “my family is harmonious,” “everyone in the family has made their own contributions to the family.” Higher score indicates a better overall family functioning. In this study, the Cronbach’s α of the family functioning questionnaire was 0.96.

The core self-evaluation scale revised by Du was used to measure student’s core self-evaluation (Du et al., 2012). The scale is of a single dimension with 10 items, of which 2, 3, 5, 7, 8, and 10 are reverse scoring questions. A 5-point scale was used, with 1 indicating “strongly disagree” and 5 indicating “strongly agree.” The higher the score, the higher the core self-evaluation level of the students. The Cronbach’s α of the scale was 0.63. Confirmatory factor analysis results demonstrated that a single-factor model fit the data satisfactorily for core self-evaluation: χ^2^/*df* = 23.16, CFI = 0.99, TLI = 0.98, RMSEA = 0.05, SRMR = 0.03, with item loadings ranging from 0.70 to 0.86.

Problematic mobile phone use was measured with the Chinese version of the Mobile Phone Problematic Use Scale (Wei et al., 2019). The scale consists of 5 aspects and 10 items. Each item is 5-point scored, from 1 (not at all) to 5 (in full accord). The higher the score, the higher the degree of problematic use of mobile phones. The Cronbach’s α of the scale was 0.86.

Loneliness was tested with the loneliness questionnaire revised by Zou (2003), which consists of 21 items in total. The questionnaire had four dimensions: pure loneliness, perception of one’s own social ability, evaluation of the current peer relationship and perception of the unsatisfied degree of important relationships. The questionnaire was scored on a 5-point scale, with 1 indicating “not at all” and 5 indicating “complete.” Except that the high score of perceived social competence represented positive evaluation, the high scores of the other three dimensions represented negative evaluation. The perceived dimension of social competence was scored reversely and then added to the scores of the other three dimensions, and the average score was taken as the total average score of loneliness. The Cronbach’s α of the scale was 0.87.

## Data Analysis

After data recovery, all invalid questionnaires (e.g., questionnaires that exceeded 20% missing values) were excluded, SPSS 25.0 and Mplus8.0 were used for statistical analysis.

## Results

### Common Method Deviation

Unrotated principal component factor analysis was performed on the items included in all variables using the Harman one-way test. The results showed that there were 8 factors with the eigenvalue greater than 1, and the variation explanation rate of the first factor was 33.79%, which was lower than the critical standard of 40% (Zhou and Long, 2004). It could be considered that there was no serious common method bias in this study.

### Descriptive Statistics and Correlation Matrix of Each Variable

The results of correlation analysis (as shown in Table 1) showed that family functioning was positively correlated with self-evaluation and gender, and negatively correlated with problematic mobile phone use and loneliness. Self-evaluation was negatively correlated with problematic mobile phone use, loneliness and gender. Problematic mobile phone use was positively correlated with loneliness, but not significantly correlated with gender. Loneliness was significantly negatively correlated with gender.

### Test of Chain Mediation Effect

**FIGURE 2:**
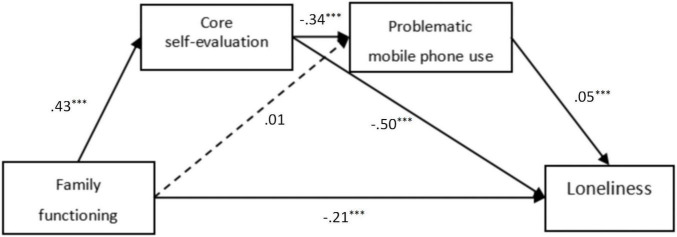
Chain mediation model. ^***^p < 0.001.

With family functioning as the independent variable, core self-evaluation and problematic use of mobile phones as the mediating variables, loneliness as the dependent variable, and gender as the control variable, the mediation model was tested (see Figure 2). [CFI = 0.99, TLI = 0.97, RMSEA (90% CI) = 0.05, SRMR = 0.02], the model fit was good. The results of path analysis showed that family functioning positively predicted core self-evaluation (β = 0.43, *p* < 0.001). Family functioning negatively predicted loneliness (β = −0.21, *p* < 0.001), and core self-evaluation negatively predicted problematic mobile phone use and loneliness (β = −0.34, *p* < 0.001; β = −0.50, *p* < 0.001). Problematic mobile phone use positively predicted loneliness (β = 0.05, *p* < 0.001). Core self-evaluation and problematic mobile phone use played a chain mediating role between family functioning and loneliness.

**TABLE 1:** Correlation analysis of family functioning, self-evaluation, problematic mobile phone use, and loneliness.

	*M* ± *SD*	1	2	3	4	5
1 Family functioning	3.91 ± 0.79	1				
2 Self-evaluation	3.36 ± 0.60	0.43**	1			
3 Problematic mobile phone use	2.84 ± 0.64	−0.14**	−0.33**	1		
4 Loneliness	2.37 ± 0.64	−0.43**	−0.61**	0.24**	1	
5 Gender		0.04**	−0.05**	0.02	−0.07**	1

**p < 0.01.

The confidence interval of the mediating effect was estimated by the bias-corrected non-parametric percentile bootstrap method. The results are shown in Table 2. The mediating effect value of core self-evaluation between family functioning and loneliness was −0.22, and the 95% confidence interval was [−0.189, −0.167]. The mediating effect of core self-evaluation was significant. The mediating effect of problematic mobile phone use between family functioning and loneliness was 0.01, and the 95% confidence interval was [−0.001, 0.002]. The mediating effect of problematic mobile phone use was not significant. Family functioning had an impact on core self-evaluation, and then on core self-evaluation affected mobile phone problem use, which finally affected loneliness. That is, core self-evaluation and problematic mobile phone use showed a chain mediating effect between family functioning and loneliness, and the mediation effect value is −0.01. The effective dose was 3%.

**TABLE 2:** Significance test of mediation effect and value of mediation effect.

Path	Effect value	Effect size	95% confidence interval	
			Lower limit	Upper limit
Family functioning →loneliness	−0.21***	60%	–0.452	–0.414
Family functioning → core Self-evaluation → loneliness	−0.22***	63%	–0.189	–0.167
Family functioning → problematic mobile phone use → loneliness	0.01	3%	–0.001	0.002
Family functioning → core Self-evaluation → problematic Mobile phone use → loneliness	−0.01***	3%	–0.008	–0.003

***p < 0.001.

## Discussion

In this study, college students from well-functioning families experienced less loneliness, which is consistent with previous studies (Johnson et al., 2001; Fujimori et al., 2017). Although the focus of interpersonal relationship among college students has shifted to peers, the family environment is still at the core of the personal social support system, and the family is still the main source of financial support and spiritual support for college students (Jiang, 2002). On the one hand, material satisfaction can relieve the realistic pressure of college students to a certain extent, such as the ability to buy high-quality services and vent negative emotions in entertainment consumption; on the other hand, college students cannot only get close emotional connection between relatives in a good family functioning environment, but also get social support from family when interpersonal relationships are frustrated. In addition, college students apply effective interpersonal communication skills acquired in a good family functioning environment to social interaction, form intimate interpersonal relationships, improve the quality of interpersonal communication (Hu et al., 2008), and further strengthen the individual’s social support system. Therefore, family, as the spiritual harbor of college students, plays an essential role in interpersonal relationships and reduces the level of loneliness.

This study found that family functioning positively predicted core self-evaluation, and core self-evaluation negatively predicted loneliness. That is, the core self-evaluation of college students played a mediating role between family functioning and loneliness. Previous studies have shown that attachment status was closely related to individual self-evaluation (Tang and Li, 2013). Good attachment relationship can promote effective communication and emotional connection between family members, thus promoting the formation of positive core self-evaluation of college students. Individuals get good self-evaluation in the family, including self-esteem, general self-efficacy and so on, which is conducive to improving the enthusiasm of individual life and learning, actively establishing interpersonal relationships, and tending to solve problems in the face of interpersonal conflicts, rather than escaping or silent treatment, thus experiencing less loneliness. Being in the situation of family dysfunction, such as the more psychological abuse and neglect an individual suffered in childhood, the easier the students form self-evaluation such as fear of rejection and lack of self-confidence, the more withdrawal and emotional inhibition they will show when establishing intimate relationships in adulthood (Yang, 2019). Therefore, the establishment of a good family functioning environment can make family members feel love and warmth, establish the concept that they are needed by others and deserved to be loved. Under the circumstance, individuals may obtain the concept of their own high sense of value, form a good self-evaluation, and then build a high-quality interpersonal network, which helps to reduce the experience of loneliness.

This study also found that family functioning positively predicted core self-evaluation and negatively predicted problematic mobile phone use, which subsequently had an impact on loneliness. This result supports the path model of mobile phone dependence, which is caused by cognitive dissonance or the need for relationship maintenance due to insecure attachment (Billieux, 2012). Family is the most primitive and core source of self-evaluation. Poor family functioning leads to low self-evaluation. Individuals with low self-evaluation tend to adopt covert, evasive and passive behaviors to maintain relationships, such as using mobile phones to meet individual emotional needs.

In this study, it is confirmed that problematic mobile phone use positively predicted loneliness, which supports the social compensation model of social media use. As the model points out that when individuals cannot get a sense of belonging in real life and lack the ability to use social resources, they will be more inclined to get help through mobile phones, networks and other tools (Park, 2003). However, the effect of using mobile phones for a long time to eliminate loneliness is not ideal, and it may cause a deeper sense of loneliness (Li, 2013; Liu et al., 2019). The possible reason is that using mobile phones for a long time not only compresses the real interpersonal emotional interaction time in reality. As a result of the deterioration of individual’s real social skills, it is easy to feel more social alienation and lack of interpersonal belonging in the real world, so loneliness will increase accordingly. At the same time, individuals tend to create a perfect interaction between themselves and others on the Internet, which results in the closure and loss of the real self and aggravates the loneliness of individuals.

The results of this study showed that family functioning had no significant effect on loneliness through problematic mobile phone use, which is inconsistent with hypothesis 2. The possible reason is that most of the research objects of the current problematic use of mobile phones are middle school students. For college students, in the case of high frequency use of mobile phones in their study, work and life, good family functioning may have a more inclusive and open attitude toward the behavior of using mobile phones. Therefore, family functioning could not have an impact on loneliness through problematic mobile phone use.

## Implications and Limitations

The results of the study confirmed that family functioning could improve individual core self-evaluation, reduce problematic use of mobile phones, thereby reducing the individual experience of loneliness. To some extent, this study makes up for the deficiency of the existing research on the family functioning of college students, and further explores the internal psychological and behavioral mechanism for the formation of loneliness, which also provides theoretical support for the intervention path to reduce the loneliness experience of college students.

This study still has the following limitations. First, the subjects are all Chinese college students, and the sample representativeness is slightly insufficient, so we can further study the subjects in different cultural backgrounds in the future. Second, the study employed the self-report method, and the survey results may have social approval effects, so we can use objective research methods such as behavior observation to collect data in the future.

## Data Availability Statement

The original contributions presented in the study are included in the article/supplementary material, further inquiries can be directed to the corresponding author/s.

## Ethics Statement

This study was reviewed by the Ethics Committee of Zhejiang Normal University. The patients/participants provided their written informed consent to participate in this study.

## Author Contributions

DW was responsible for guiding the structure of the article. MJ was responsible for data analysis. WW and CN were responsible for English revision. All authors listed have made a substantial, direct, and intellectual contribution to the work, and approved it for publication.

## Conflict of Interest

The authors declare that the research was conducted in the absence of any commercial or financial relationships that could be construed as a potential conflict of interest.

## Publisher’s Note

All claims expressed in this article are solely those of the authors and do not necessarily represent those of their affiliated organizations, or those of the publisher, the editors and the reviewers. Any product that may be evaluated in this article, or claim that may be made by its manufacturer, is not guaranteed or endorsed by the publisher.
